# Reciprocal Modulation of Surface Expression of Annexin A2 in a Human Umbilical Vein Endothelial Cell-Derived Cell Line by Eicosapentaenoic Acid and Docosahexaenoic Acid

**DOI:** 10.1371/journal.pone.0085045

**Published:** 2014-01-21

**Authors:** Jungha Park, Takayuki Yamaura, Jun Kawamoto, Tatsuo Kurihara, Satoshi B. Sato

**Affiliations:** 1 Research Center for Low Temperature and Materials Sciences, Kyoto University, Kyoto, Kyoto, Japan; 2 Institute for Chemical Research, Kyoto University, Uji, Kyoto, Japan; Max Delbrueck Center for Molecular Medicine, Germany

## Abstract

**Background:**

Annexin A2 (ANXA2), a member of the annexin family of cytosolic Ca^2+^-binding proteins, plays a pivotal role in vascular biology. Small amounts of this protein and S100A10 protein are exposed on the surface of endothelial cells (ECs). They control fibrinolysis by recruiting tissue-type and urokinase-type plasminogen activators from the plasma. Nutritional studies indicate that two major long-chain polyunsaturated fatty acids (PUFAs), i.e., eicosapentaenoic acid (EPA) and docosahexaenoic acid (DHA), provide benefits for EC functions. The effects of EPA and DHA on the plasminogen/plasmin system have not been characterized.

**Methodology/Principal Findings:**

Proteomic analysis of a cultured human umbilical vein EC-derived cell line, HUV-EC-C, showed that cell-associated ANXA2 decreased with EPA treatment and increased with DHA. A small fraction of ANXA2 was bound to the cell surface, which was also affected by these PUFAs following the same trends. Cell surface expression was negatively regulated by protein kinase C (PKC) α-mediated Ser-phosphorylation, which was up- and down-regulated by EPA and DHA, respectively. These PUFAs differentially affected a small fraction of caveolae/rafts-associated ANXA2. In addition to chymotrypsin-like activity in the serum, newly activated plasmin cleaved the ANXA2 on the cell surface at distinct sites in the N-terminal sequence. ANXA2 also bound to membranes released in the medium, which was similarly processed by these proteases. Both the PUFAs did not directly affect the release.

**Conclusion/Significance:**

These results suggest that EPA and DHA reciprocally control cell surface location of ANXA2. Moreover, cleavage of this protein by plasmin likely resulted in autodigestion of the platform for formation of this protease. In conjunction with termination of the proteolysis by rapid inactivation of plasmin by α-2-antiplasmin and other polypeptide inhibitors, this feedback mechanism may emphasize the benefits of these PUFA in regulation of the initiation of fibrinolysis on the surface of ECs.

## Introduction

Vascular endothelial cells (ECs) manifest both the progression and recovery phases of vascular lesions. ECs express a large repertoire of receptor tyrosine kinases (RTKs) and G-protein coupled receptors (GPCRs) for inflammatory or angiogenic ligands [1], [2]. These ligands control various aspects of activities of ECs, such as vasoconstriction, dilation, inflammation and angiogenesis. Multiple proteolytic reactions occur on the surfaces of ECs, modulating various aspects of the cellular environment. Of these reactions, the plasminogen/plasmin system, which cleaves and activates plasminogen through tissue and urokinase-type plasminogen activators (tPA and uPA, respectively) is primarily important for fibrinolysis and control of inflammation. In this system, participation of annexin A2 (ANXA2) is critical [3]. A very small amount of this protein, which is originally distributed in the cytoplasm, is exposed on the cell surface as a heterotetramer with S100A10 (also called p11). This complex binds all the elements in the plasminogen/plasmin system [3], [4]. While other membrane proteins in various cell types can also bind plasminogen, ANXA2 plays the primary role in fibrinolysis. This has been well demonstrated by analysis of *AnxA2*-deficient mice, in which fibrin was found to accumulate within the lungs and many other organs [6].

ANXA2 is believed to first bind to acidic phospholipids on the cytoplasmic face of the membrane when the intracellular concentration of Ca^2+^ increases. Although the mechanism is not fully shown, protein phosphorylation reactions in RTK signaling pathways regulate the surface expression of ANXA2 [7], [8]. ANXA2 has been suggested to translocate from the cytoplasm to the cell surface in ECs [7]. In non-endothelial cells, ANXA2 can be transported by vesicular dynamics [9]. ANXA2 is also expressed in immune cells such as neutrophils and macrophages. Plasmin degrades and modifies the extracellular matrix (ECM). In conjunction with membrane-type 1 matrix metalloproteinase, ANXA2 further amplifies the degradation of ECM through cleavage and activation of pro-matrix metalloproteinase-2 [10]. ANXA2 is also expressed on the surface of several types of cancer cells and plays a role in migration and invasion [11]. Furthermore, ANXA2 serves a binding site for β_2_-glycoprotein I on ECs and immune cells. Autoantibodies bind to β_2_-glycoprotein I in antiphospholipid syndrome, a disorder that is associated with the increased incidence of venous and arterial thrombosis [12].

Dietary intake of the major long-chain omega-3 polyunsaturated fatty acids (PUFAs), eicosapentaenoic acid (EPA, 20∶5^Δ5, 8, 11, 14, 17^) and docosahexaenoic acid (DHA, 22∶6^Δ4, 7, 10, 13, 16, 19^), reduces the risk of cardiovascular diseases [13]–[19]. These PUFAs activate the nuclear receptors, PPARα and PPARγ, thereby downregulating the expression of NF-κB [20]–[22]. Metabolically, EPA can be converted to a non-functional compound that antagonizes the production of proinflammatory cytokines and formation of inflammatory eicosanoids from arachidonic acid [2], [23]. EPA and DHA are also converted into the inflammation-resolving mediators, resolvins and protectins [23], [24]. EPA and DHA activate endothelial NO synthase (eNOS) by inducing their delocalization from caveolae [25], [26]. Therefore, EPA and DHA might mediate relaxation of vascular smooth muscle cells through release of NO. Whether these PUFAs affect elements in fibrinolysis has not been reported.

Human umbilical vein endothelial cells (HUVECs) have often been used as *in vitro* models to characterize molecular mechanism underlying EC function. Although the properties of HUVECs are not representative of those of all ECs, proteomic analyses have been conducted to characterize their basic protein profile as well as toxicological responses [27]–[30]. HUV-EC-C, a cell line with a limited life-span, is derived from HUVECs [31]. In this study, we used this cell line to analyze the effects of EPA and DHA on membrane events. The use of a cell line was important for avoiding variation in cell surface properties caused by the relatively harsh proteolysis conditions needed to disperse primary HUVECs. In this cell line, ANXA2 was expressed on the surface and was proteolytically processed near the N-terminus by chymotrypsin-like serine protease and plasmin. ANXA2 was also released in a membrane-bound form, which was similarly processed by chymotrypsin-like enzyme. While these events may induce digestion of the ANXA2/S100A10 platform and thus facilitate the termination of fibrinolysis, EPA and DHA negatively and positively modulated binding of ANXA2 to the live cell surface. This was likely controlled through up- and down-regulation of inhibitory Ser-phosphorylation of ANXA2, respectively. Our results suggest that EPA and DHA reciprocally regulate the initiation of fibrinolysis on the surface of ECs.

## Results

### EPA and DHA affected the expression of ANXA2 in HUV-EC-C

We analyzed the effects of EPA and DHA on ANXA2 expression in the HUV-EC-C cells line using proteomics methods. The cells were maintained in medium 200 plus Low Serum Growth Supplement (LSGS) and 8% heat-inactivated fetal bovine serum (FBS). To wait for the recovery of surface property after trypsin/EDTA-treatment, the cells were used 72 h after splitting. The cells were treated with PUFAs without the heat-inactivated FBS to eliminate the effect of denatured protein and for analysis of the culture supernatant. The concentration of PUFA was 10 µM, which was 1/10th to 1/45th of the free fatty acid level in sera of healthy adults.

**Figure 1:**
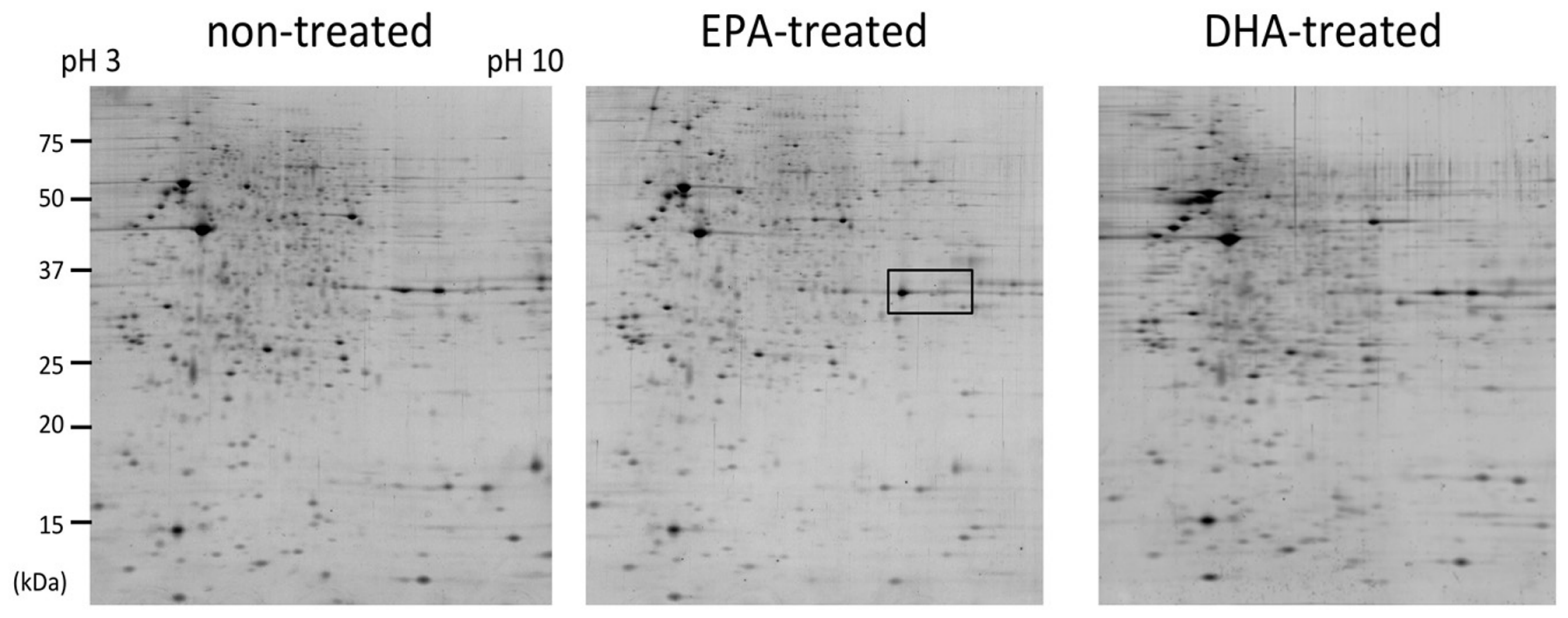
Two-dimensional gel electrophoresis of HUV-EC-C cells (left) and those treated with 10 µM EPA (middle) or DHA (right) for 24 h. Cell extracts were separated in the 1st dimension with an immobilized proton gradient strip (pH between 3.0 and 10.0). EPA treatment reduced the intensity of spots distributing at 36 kDa, at pH around 7 (surrounded in a rectangle). The intensity of a spot with the same molecular mass increased in DHA-treated cells. Peptide mass fingerprinting analysis identified these spots as annexin A2.

Using 2D-electrophoresis (isoelectric focusing at pH between 3.0 and 10.0 and subsequent SDS-PAGE [32]), we found that spots at 36 kDa were distributed differently in extracts from EPA- and DHA-treated cells (Fig. 1). Oleic acid (OLA) treatment showed no effect on the distribution of proteins in this area of the gel (data not shown). Analysis by peptide mass fingerprinting using MALDI-TOF indicated that these spots corresponded to ANXA2. Proteins with different isoelectric points likely contained different posttranslational modification such as phosphorylation. Further systematic analysis identified other proteins affected by EPA and DHA treatment (data not shown). We chose to characterize ANXA2 initially because of its primary importance in vascular biology.

### EPA and DHA also reciprocally affected ANXA2 bound to the cell surface

**Figure 2:**
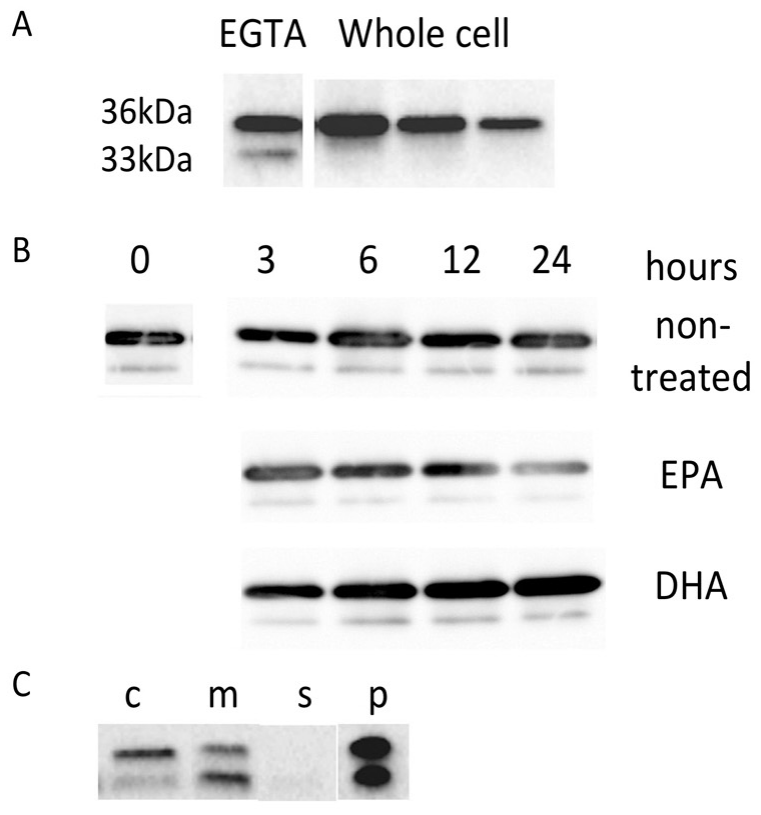
The presence of ANXA2 on the cell surface (A), the effect of EPA and DHA on this protein (B), and the release of ANXA2-bound membranes in the culture medium (C). (A) Left; Surface-bound ANXA2 was extracted in Hank's-EGTA. One-fiftieth of the total amount was examined by immunoblotting. Two bands at 36 kDa and 33 kDa were detected. Right; serial dilution of a whole lysate of the same cell after treatment with Hank's-EGTA was analyzed. Only 36 kDa was present. The numbers denote fold-dilution of the original amount. (B) Cells were washed and incubated with a new culture medium containing EPA or DHA. At indicated time, surface-bound ANXA2 was extracted in Hank's-EGTA. While the amount in non-treated cells (upper lane) was constant, it was decreased by EPA (10 µM, middle lane) and increased by DHA (lower lane). (C) ANXA2 was not only present on the surface (c) but also in the culture supernatant (m, s, p). The same portions of Hank's-EGTA and the supernatant after centrifugation at 2,000×g for 5 min (m) and additionally at 17,000×g for 30 min (s) were analyzed. All 36 kDa and most of 33 kDa were sedimented by the last centrifugation (p, 10-fold concentrated).

For analysis of cell surface-bound ANXA2, cells were treated with Hank's-EGTA at 4°C, which blocked Ca^2+^-dependent binding to acidic phospholipids [7], [33]. Cell viability was minimally affected by this treatment. Release of lactate dehydrogenase was only 0.52%±0.03% of the total activity (n = 2). ANXA2 was detected by immunoblotting performed using a commercially available rabbit monoclonal antibody that binds to residues surrounding Phe307 near the C terminus of human ANXA2. Two protein bands at ca. 36 kDa and ca. 33 kDa (hereafter referred to as 36 kDa and 33 kDa, respectively) were present in the EGTA extract, which had been centrifuged at 2,000×g for 5 min prior to analysis (Fig. 2A). They remained in the supernatant after centrifugation at 17,000×g for 10 min, and even 100,000×g for 1 h (data not shown). In contrast, only 36 kDa was present in the whole cell lysate prepared after treatment with EGTA (Fig. 2A). By analysis of a serial dilution of these extracts, the amount of 36 kDa released by EGTA was estimated to be 1.0%±0.4% of the total amount (n = 3). It was likely that surface-bound ANXA2 was cleaved near the N-terminus to generate 33 kDa. In contrast to the results for ANXA2, we did not detect S100A10 in the extract by immunoblotting (data not shown). Unlike other S100 proteins, S100A10 binds to membranes independently of Ca^2+^ [34].

When cells were chased in a medium containing EPA, the amount of surface-bound ANXA2 gradually decreased (Fig. 2B). At 24 h, the level of 36 kDa was reduced to nearly 50% of that in non-treated cells, while 33 kDa was barely detectable. In contrast, DHA gradually enhanced surface expression of 36 kDa, which was nearly 50% higher than the amount in non-treated cells (Fig. 2B). The amount of 33 kDa also gradually increased. These results suggest that EPA and DHA also reciprocally affected the cell-surface location of ANXA2. Because the entire changed amount of ANXA2 in the whole cell lysate appeared to be larger than the changed amount on the surface, EPA and DHA might have changed other populations of this protein, e.g., those distributed in the nucleus [35], [36]. While ANXA2 can also play roles in such places, we focused exclusively on the fraction exposed in the medium.

### Membrane-bound ANXA2 was also released in the medium

We found that the 36 kDa and 33 kDa forms of ANXA2 were also present in the culture supernatant that had been centrifugation at 2,000×g for 5 min. Although the amount of each protein varied frequently among different cultures (data not shown), it was roughly comparable to that in the EGTA extract (Fig. 2C, m). Both proteins were sedimented after centrifugation at 17,000×g for 30 min, with only a very small amount of 33 kDa remaining in the supernatant (Fig. 2C, p, s). This protein was not sedimented by additional centrifugation at 100,000×g for 1 h (data not shown). These results suggested that ANXA2 was released from the cells in a form bound to free membranes. The effects of EPA and DHA on this population of ANXA2 were also studied (see below).

### Cell surface-bound and released forms of ANXA2 were cleaved by a chymotrypsin-like enzyme and plasmin

**Figure 3:**
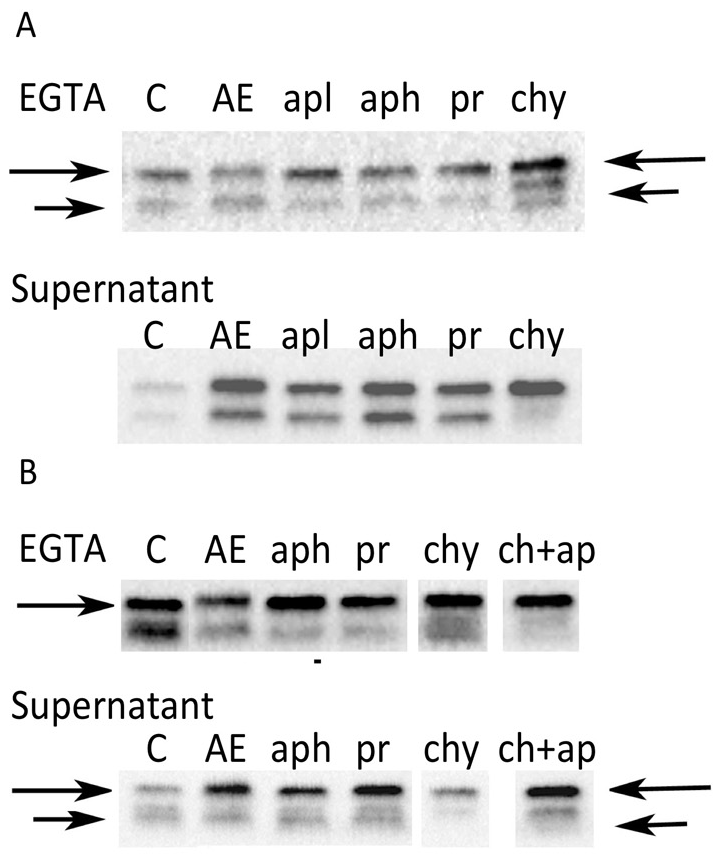
Effect of protease inhibitors on the cell-surface bound and the released ANXA2. (A) Cells were treated with none (c), AEBSF (AE, 50 µM), α-2-antiplasmin (0.33 µM; apl, 0.66 µM; aph), aprotinin (pr, 1.3 µM) and chymostatin (chy, 30 µM) for 24 h. The same portions of Hank's-EGTA extracts (upper lane) and culture supernatants after centrifugation at 2,000×g for 5 min (lower lane) were analyzed. Longer arrows indicate the position of 36 kDa whereas shorter ones indicate that of 33 kDa that was present in non-treated cells. In the presence of chymostatin, formation of 33 kDa was blocked but two new bands were present. (B) Cells were treated with Hank's-EGTA in advance and cultured in the presence of indicated inhibitors. The concentration of α-2-antiplasmin was 0.66 µM (aph). Cells were also treated with this concentration of α-2-antiplasmin and chymostatin (ch + ap). After 24 h, ANXA newly extracted in Hank's-EGTA (upper lane) and in culture supernatant (lower lane) were analyzed as in (A). Other reagents were used at the same concentration as in (A). Arrows indicate the positions of 36 kDa and 33 kDa as in (A).

We tried to identify the site that generated 33 kDa on the cell surface and the released membranes. The N-terminal sequence of ANXA2 contains three trypsin-cleavable sites (Lys9, Lys27 and Lys45) and one chymotrypsin-cleavable site (Phe32). We added various serine protease inhibitors to the culture (Fig. 3A). The cell viability was not affected by these inhibitors (not shown). Two inhibitor peptides of plasmin, i.e., α-2-antiplasmin (0.33 and 0.66 µM, abbreviated to as apl and aph, respectively, in Fig. 3A) and aprotinin (pr, ca. 1.3 µM), and a small molecule inhibitor, 4-(2-aminoethyl) benzensulfonyl fluoride (AEBSF or AE, 50 µM), scarcely affected the amounts of 36 kDa and 33 kDa (Fig. 3A). In contrast, chymostatin treatment (chy, 30 µM) remarkably inhibited the formation of 33 kDa and resulted in two new bands that were approximately 3 kDa and 5 kDa smaller than 36 kDa (i.e., ca. 33 kDa and ca. 31 kDa, Fig. 3A). The upper band was larger than 33 kDa that was initially found in EGTA extract. These results strongly suggested that the firstly-found 33 kDa was generated by cleavage by a chymotrypsin-like enzyme at Phe32. In the absence of this activity, ANXA2 was likely cleaved by plasmin at Lys27 and Lys45. With the present method, it is not clear if ANXA2 was cleaved at Lys9.

Chymostatin in the aqueous phase is short-lived due to inactivation of the reactive aldehyde group. Therefore, chymotrypsin-like activity in the medium was most likely present in the aqueous phase at the start of the experiment, but was not secreted by the cells during the chase. A serum used in this study and also a stock of fetal bovine serum contained a very low but significant chymotrypsin-like activity that hydrolyzed a colorimetric substrate, N-Succinyl-Ala-Ala-Pro-Phe p-nitroanilide (data not shown). This activity might have been released from immune cells during preparation (see Discussion). In contrast, HUVECs readily secrete tPA that binds to ANXA2 [37]. Plasminogen as well as pro-uPA was likely present in LSGS supplemented to the culture medium. These results suggested that ANXA2 on the cell surface was not only functionally interacted with the plasminogen/plasmin system but also served a substrate for the activated enzyme. The low level of plasmin-mediated digestion was likely due to the low serum content (2%).

When culture supernatants were analyzed, α-2-antiplasmin, aprotinin and AEBSF again did not inhibit the formation of 33 kDa (Fig. 3A, lower lane). In the presence of chymostatin, formation of 33 kDa was prevented, while the two new proteins that were found on the cell surface were scarcely detected. These results indicated that released membrane-bound ANXA2 was similarly processed by the chymotrypsin-like enzyme but less efficiently by plasmin. The difference was likely due to low binding efficiency of tPA to ANXA2 secreted from the cells. It was also found that these protease inhibitors increased the released amount of 36 kDa. Inhibitors of plasmin further increased the amount of 33 kDa. These results suggested that plasmin-mediated proteolysis upregulated release of the ANXA2-bound membranes.

### Newly appeared ANXA2 on the cell surface was also processed by proteases

Plasmin, tPA, uPA and plasminogen can bind to the ANXA2/S100A10 at distinct sites (see ref. [3]–[5]). The effects of these proteins were removed by extracting ANXA2 by EGTA in advance. After culturing in fresh medium for 24 h, newly appeared ANXA2 was examined by an additional EGTA treatment (Fig. 3B). Both 36 kDa and 33 kDa were absent at the start of the experiment (data not shown). However, these proteins reappeared on the surface at levels similar to those seen prior to EGTA treatment. These results indicated that a new round of surface expression of ANXA2 occurred without pre-located ANXA2.

In contrast to such similarity, the pattern of subsequent proteolytic processing was different. In control cells, the proteolysis yielded a thick diffuse band at 33 kDa. In the presence of plasmin inhibitors, the band became sharp and weak. In the presence of chymostatin, the band also became weak but remained diffuse. Moreover, a thin band appeared at around 31 kDa. When the cells were treated with chymostatin in conjunction with α-2-antiplasmin, the bands except for 36 kDa became very faint (Fig. 3B, ch + ap). These results suggested that both plasmin and chymotrypsin-like enzyme contributed to the formation of the diffuse band. While yet undefined factor may also have contributed to the formation of diffuse band, further speciation was not done in this study. However, these results indicated that newly appeared ANXA2 also functionally interacted with the plasminogen/plasmin system.

### Plasmin inhibitors enhanced the release of membrane-bound ANXA2 from EGTA-pretreated cells

When the culture supernatant was analyzed, both 36 kDa and 33 kDa were present (Fig. 3B, lower lane). Inhibitors of plasmin again enhanced the release of the membrane-bound 36 kDa while chymostatin slightly reduced the release. When cells were treated with α-2-antiplasmin and chymostatin, the release was enhanced. These results indicated that inhibition of plasmin dominantly enhanced the release of membrane-bound ANXA2. The patterns of processing of released ANXA2 were slightly different from those seen prior to EGTA treatment. In the absence of inhibitors, two bands distributed at around 33 kDa. The combination of α-2-antiplasmin and chymostatin increased the amount a protein at upper position. Although much weaker, this band was also present in cells singly treated with plasmin inhibitors. This biased proteolysis of ANXA2 suggested that non-plasmin trypsin-like activity participated in the early processing of membrane-bound ANXA2.

### EPA and DHA reciprocally changed cell surface-binding of ANXA2 but not substantially affected its release in the medium

**Figure 4:**
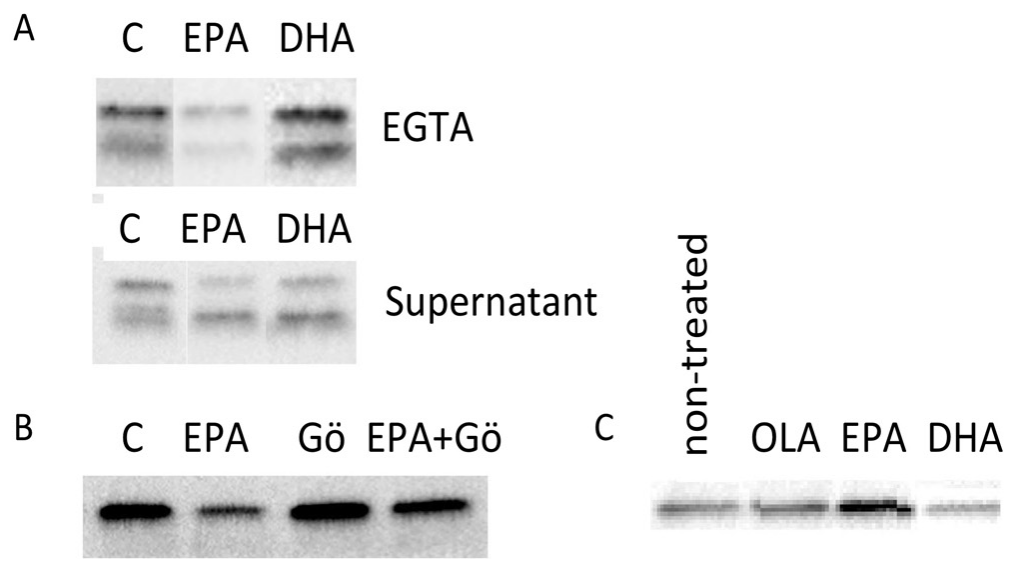
Effects of EPA, DHA and Gö6976 on ANXA2 on the cell surface and in culture supernatant, and on Ser-phosphorylation of ANXA2. Cells were pretreated with Hank's-EGTA and examined after culturing for 24 h. (A) EPA (10 µM) reduced, and DHA (10 µM) increased the amount of 36 kDa and also 33 kDa in the EGTA extract (upper lane). In the culture supernatant (lower lane), these PUFAs marginally reduced the amount of 36 kDa. (B) The cells were incubated EPA and Gö6976 (50 nM). Gö6976 increased the surface-bound 36 kDa. It also suppressed the effect of EPA. (C) Effects of EPA, DHA and OLA on Ser-phosphorylation of ANXA2. The same protein amounts of whole extract of cells treated with indicated fatty acids (10 µM) were incubated with anti-phosphoserine mAb and the same portions of immunoprecipitates were examined for ANXA2. EPA increased the amount of the Ser-phosphorylated ANXA2. In contrast, DHA slightly reduced the amount.

Having identified multilayered controls of ANXA2 processing by serine proteases, we addressed the effect of EPA and DHA on cells pretreated with EGTA. EPA remarkably suppressed the new round of appearance of ANXA2 on the cell surface (Fig. 4A, upper lane). In contrast, DHA increased the surface expression of ANXA2 (Fig. 4A). The amount of 33 kDa had also increased, suggesting that DHA did not inhibit proteolysis. These results indicated that reciprocal control of cell surface-binding of ANXA2 by these PUFAs occurred regardless of the preoccupation of ANXA2. In contrast, both EPA and DHA only marginally reduced the released of 36 kDa and did not sizably change that of 33 kDa in the medium (Fig. 4A, lower lane). Minor inhibition by EPA suggested that the release of ANXA2-bound membranes was regulated independently of the cell surface-location mechanism of ANXA2.

### Effect of EPA was suppressed by an inhibitor of PKCα

Cell-surface binding of ANXA2 in primary EC cultures is negatively regulated by Ser-phosphorylation by PKCα [8]. We assessed effect of the PKCα inhibitor, Gö6976 (50 nM) on the surface expression of 36 kDa. In agreement with the published result [8], we found that this reagent slightly increased the amount of surface-bound ANXA2 in EGTA-pretreated cells (Fig. 4B). It also suppressed the effect of EPA on the downregulation of surface-bound ANXA2 (Fig. 4B). These results suggested that EPA likely upregulated basal regulation by PKCα.

### EPA upregulated while DHA downregulated Ser-phosphorylation of ANXA2

We assessed the effect of EPA on Ser-phosphorylation of ANXA2. Because the fraction of cell surface-bound ANXA2 was small, Ser-phosphorylated proteins were first enriched by immunoprecipitation, followed by probing for ANXA2 as previously described [8]. ANXA2 in the control cells was basally phosphorylated (Fig. 4C). Ser phosphorylation of ANXA2 was slightly but definitely enhanced with EPA treatment, while this effect was reduced by DHA. OLA treatment did not induce a change in ANXA2 phosphorylation. The increase in the phosphorylation by EPA was 15–30% of the control, while the decrease induced by DHA was 5–10% (n = 3).

### EPA and DHA did not change distribution of NXA2 in detergent-insoluble fraction

**Figure 5:**
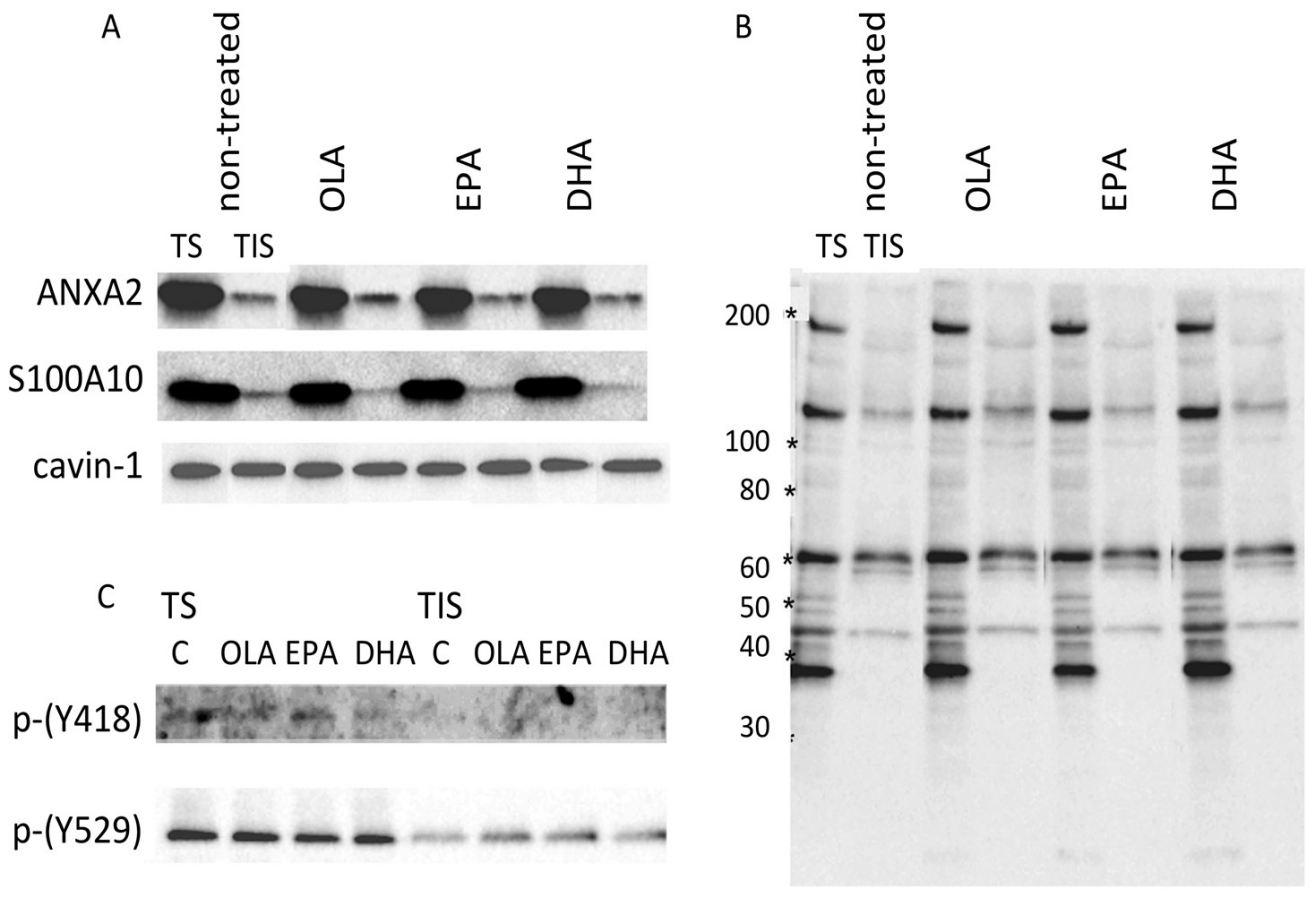
Effects of EPA, DHA and OLA on the distribution of ANXA2, S100A10 and cavin-1 in Triton X-100-soluble (TS, 2.6% of the total volume) and insoluble (TIS, 1.3% of the total volume) fractions (A), on Tyr-phosphorylated proteins analyzed by using anti-phosphotyrosine mAb (B) and on Src that was phosphorylated either at Tyr418 and Tyr529 (C). Proteins were analyzed by immunoblotting. EPA and DHA did not affect the amounts of ANXA2, S100A10 and cavin-1 in the TIS fraction (A). The intensities of Tyr-phosphorylated protein at 36 kDa were different in the presence of EPA and DHA (B). Very small amounts of p-Src(Y418) were present only in the TS fraction. p-Src(Y529) was present in both fractions with enrichment in the TS fraction regardless of the treatments with EPA and DHA.

Many aspects of receptor and Ca^2+^-dependent signaling are linked to caveolae/lipid rafts. We investigated whether EPA and DHA affected the distribution of ANXA2 in such membrane domains by analyzing Triton X-100-soluble (TS) and insoluble (TIS) fractions (Fig. 5A). In the TIS fraction, 11%±2% of the total amount of ANXA2 was present. Both EPA and DHA did not sizably change their amounts. The amount of S100A10 in the TIS fraction was also similar with both PUFAs. The distribution of cavin-1, a regulator of caveola formation that is present both in cytoplasm and caveolae [38], also did not change in the TIS fraction. These results suggested that EPA and DHA minimally affected the integrity of caveolae/rafts and also the average mode of association of ANXA2.

### EPA and DHA did not substantially affect tyrosine- phosphorylated proteins in both the TS and TIS fractions

Phosphorylation on Tyr23 is implied to be relevant to the cell surface as well as nuclear localization of ANXA2 [5], [7]. We compared patterns of Tyr-phosphorylated proteins in the TS and TIS fractions by immunoblotting (Fig. 5B). Distribution of phosphorylated proteins was distinct between these fractions. A 36-kDa band was present in the TS but not the TIS fraction. While the intensity of other individual bands were not sizably changed following treatment with either EPA or DHA, the intensity of the 36-kDa band was slightly reduced by EPA and increased by DHA (Fig. 5B). We, however, could not confirm identity of the protein in TS to be ANXA2 because Tyr- phosphorylated proteins could not be enriched by immunoprecipitation with the mAb used for detection by immunoblotting (data not shown).

Src Tyr kinase has been implicated in Tyr-phosphorylation of ANXA2 [7], [8]. Human Src is inactivated by phosphorylation of Tyr529 and activated by binding to SH2 and SH3 domain in conjunction with phosphorylation of Tyr418. We examined phosphorylation on these residues by immunoblotting. Very small amounts of p-Src(Y418) were present, and only in the TS fraction. Treatment with either EPA or DHA exerted no effect (Fig. 5C). p-Src(Y529) was present in both fractions, with enrichment in the TS fraction. The amount of this protein in TIS was slightly higher in cells treated with these PUFAs, but also after treatment with OLA.

While no substantial effect of these PUFAs on Src was apparent, distribution of p-Src(Y418) implied that ANXA2 might be Tyr-phosphorylated only outside of caveolae/rafts. It either remained excluded from the domain, or was rapidly dephosphorylated after translocation.

### EPA and DHA affected ANXA2 in a small specific population of caveolae

**Figure 6:**
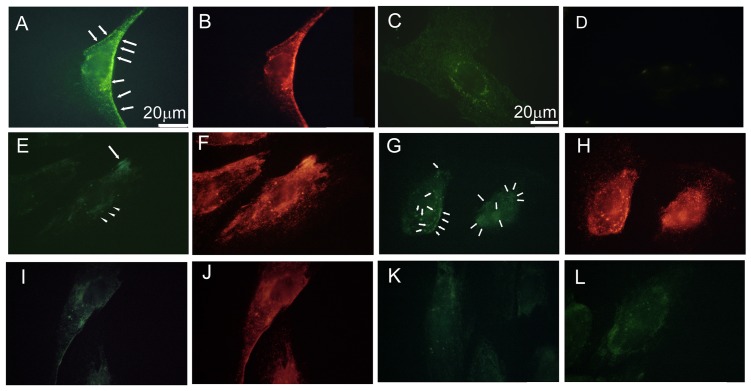
Immunofluorescence analysis of ANXA2, caveolin-1 and eNOS. Cells were treated with EPA or DHA (10 µM) for 24 h. (A), (B) Immunofluorescences of ANXA2 (A) and caveolin-1 (B) in the permeabilized non-treated cells were colocalized to punctate structures. Arrows in (A) indicate ANXA2 accumulated at the peripheral caveolar arrays. (C) Cell-surface distribution of ANXA2 in the non-permeabilized cell. (D) The immunofluorescence was lost when cells were pretreated with EGTA. (E), (F) Distribution of ANXA2 (E) and caveolin-1 (F) after EPA-treatment. Only weak immunofluorescence of ANXA2 was localized to perinuclear vesicles (arrow heads) and a peripheral cluster (arrow). Caveolin-1 immunofluoresce remained strong, which localized to these compartments and other structures distributing in the cytoplasm. (G), (H) Distribution of ANXA2 (G) and caveolin-1 (H) in DHA-treated cells. ANXA2 and caveolin-1 were localized to vesicular compartments near and above the nucleus (arrows). (I), (J) Distribution of eNOS (I) and caveolin-1 (J) in non-treated cells indicated colocalization in caveolar compartments. Immunofluorescence of eNOS in EPA (K) and (L) DHA-treated cells, respective, were very weak.

The cell fractionation results raised a possibility that DHA and EPA might affect a limited population of ANXA2. We directly studied its distribution in HUV-EC-C by immunofluorescence microscopy (Fig. 6). Many of the non-treated cells showed a morphology that was consistent with migration. Weak ANXA2 immunofluorescence was localized to small vesicles near and along the rear edge and to those distributing near above the nucleus (Fig. 6A, arrows). The distribution pattern was similar to that of caveolin-1 (Fig. 6B). Without cell permeabilization, weak immunofluorescence was distributed over the entire surface and in some spots near the nucleus (Fig. 6C). When the cells were treated with EGTA before fixation, immunofluorescence was lost (Fig. 6D). These results suggested that ANXA2 was accumulated on the cytoplasmic face of plasma-membrane bound caveolae that were accumulated cell periphery and near above the nucleus, while it spread over the cell surface.

In the presence of EPA, the cell morphology did not indicate migration. ANXA2 immunofluorescence was no longer localized to a line of dots. It was, however, localized to a cluster of vesicles near the cell periphery and to perinuclear Golgi-like stacks (Fig. 6E, arrows and arrow heads, respectively). These compartments colocalized to caveolin-1 immunofluorescence. However, most of caveolin-1 immunofluorescence distributed in a punctate pattern without ANXA2 (Fig. 5F). This result suggested that caveolin-1 and ANXA2 were no longer localized to the caveolar arrays on the cell periphery and near the nucleus. As with EPA, the morphology of DHA-treated cells also did not show migration. In contrast to EPA, ANXA2 and caveolin-1 were colocalized to arrays of vesicles distributing near the nucleus (Fig. 5G, H).

eNOS has been shown to interact with caveolin-1. EPA and DHA activate this protein by inducing its delocalization from caveolae [25], [26]. We observed a weak but clearly detectable eNOS immunofluorescence with caveolin-1 (Fig. 6I, J). In contrast, eNOS was delocalized from caveolae by both PUFAs (Fig. 5K, L).

These results suggest that EPA and DHA distinctively affected the distribution of ANXA2-associated caveolae. In contrast, both PUFAs delocalized eNOS from caveolae. ANXA2 binds to membrane surface by annexin repeat [5] while eNOS does by in-bilayer distribution of one myristoyl and two palmitoyl chains that covalently bound to the residues in the near N terminus [39]. The differential effects of EPA and DHA on these proteins might be due to these structural properties.

## Discussion

Nutritional and epidemiological studies have shown that EPA and DHA in fish oil can decrease the risk of cardiovascular diseases [13], [19]. To find a biological basis for this, we used proteomics methods to examine the effect of pure forms of these PUFAs on a HUVEC-derived cell line. The results showed that EPA and DHA induced reciprocal changes in the expression of ANXA2 (Fig. 1). Subsequent analysis demonstrated that these PUFAs also affected the cell-surface bound ANXA2, thereby following the same trend (Fig. 2). While ANXA2 has been shown to play roles in the nucleus [33], [34], its function as an interface between cytoplasmic signaling events and the plasminogen/plasmin system are well known in vascular biology and cancer studies. In the present study, we focused on this membrane-bound fraction of ANXA2.

ANXA2 was processed to a 33-kDa form on both the cell surface and on the released membranes. Since this protein was absent in the cytoplasm (Fig. 2), it was likely that ANXA2 could only make contact with protease(s) in the extracellular space. The use of specific protease inhibitors strongly suggested that a chymotrypsin-like enzyme, which was likely present in the serum supplement, cleaved the N-terminal sequence at Phe32 (Fig. 3). The serum growth supplement, and also FBS used in this study contained low but significant chymotrypsin-like activity. This activity may have been generated during the isolation of serum from blood. However, it may not be merely a contaminant, since chymotrypsin-like enzymes are occasionally released and even circulate in the blood flow during inflammation. For example, chymases from mast cells and cathepsin G from neutrophils control inflammatory responses [40], [41]. Chymases are also released from placenta, inducing the expression of various cell adhesion molecules in human uterine microvascular endothelial cells [42]. Remarkably, we found that chymostatin did not completely prevent proteolysis of ANXA2. Rather, ANXA2 was partially processed, yielding products presumably cleaved at Lys27 and Lys45. This was almost completely prevented by a combination of chymostatin and α-2-antiplasmin (Fig. 3). Our results suggested that plasmin could mediate limited proteolysis in the absence of chymotrypsin-like enzymes. The concentrations of plasminogen in normal human and fetal bovine sera are both approximately 1.4 µM [43], [44], while the concentration in the human conceptus is ≤50% of that found in maternal serum [45]. tPA is secreted from primary cultures of HUVEC without stimulation [37]. Although the concentration of plasminogen was 50-fold lower in the present study, tPA, and also uPA, could cleave and activate plasminogen on the cell surface when ANXA2 was present. Although we did not directly rule out the binding of active plasmin in the serum supplements, the concentration of inhibitors of plasmin, e.g., anti-trypsin, α2-macroblobulin was higher than that of plasmin in the human conceptus [45]. The serum may also contain plasminogen activator inhibitor-1 (PAI-1), and also PAI-2. The supplemented concentration of α-2-antiplasmin in this study was 10-fold higher than the estimated concentration of plasminogen.

Regardless of the pretreatment with EGTA, we showed that ANXA2-bound membranes were released in the medium (Fig. 3, 4). On the released membrane, ANXA2 was processed by chymotrypsin-like enzyme and less efficiently by plasmin. The released ANXA2 fraction may have a wider distribution or may even circulate in the bloodstream. A potential mechanism for this release is exocytic discharge of intracellular vesicles. Exocytosis of ANXA2-bound rafts in endosomal multivesicular bodies has been shown [9]. A similar or related mechanism has been implied in the release of vesicles. For example, a small fraction of microvesicles derived from monocytes/macrophages contain tissue factor [46]. While they appear to be used for thrombosis, the remaining fractions presumably contain other proteins. Tumor necrosis factor α (TNFα)-stimulated ECs, which eventually promote apoptosis, release uPA-bound vesicles as microparticles that cleaves and activates plasminogen [47]. Notably, many of these membranes are implied to be released under pathologic conditions. In this study, ANXA-bound membranes might be released by a certain pathologic stimulus. Since membranes released from such cells are often heterogeneous, it will be necessary to analyze the biochemical properties of individual carrier membranes to further elucidate the physiological significance of this fraction of ANXA2.

Two outcomes of processing by plasmin and chymotrypsin-like enzyme(s) are apparent. First, these proteases could decrease the number of ANXA2/S100A10 complexes. The 11-amino-acid N-terminal sequence of ANXA2 is essential for interaction with S100A10 [34]. The proteases analyzed in this study could readily dissociate the tetramer by removing this residue. This negative regulation mechanism may prevent chronic activation of plasmin and other enzymes. Proteolysis of surface-bound ANXA2 and dissociation of the heterotetrameric complex have been shown in monocytes [48]. Analysis with a recombinant ANXA2 (K27A) suggested that the protease responsible for this was a trypsin-like enzyme [48]. Taking present results into account, it is likely that plasmin itself is involved in the downregulation of plasmin-mediated chemotaxis of these cells.

Processing of cell surface proteins by plasmin may also specifically control the release of ANXA2-bound membranes. This was suggested by the differential effects of chymostatin and α-2-antiplasmin on the release (Fig. 3). In the presence of chymostatin, when only plasmin was active, the release of ANXA2 from cells to the medium did not change substantially. This was in contrast to the enhanced release in the presence of plasmin inhibitors. In addition to degradation and modification of ECM and activation of several matrix metalloproteinase zymogens, plasmin can also induce release of growth factors and cytokines from the stroma [49]. Moreover, plasmin cleaves transmembrane molecules of the viable cells, thereby generating functionally important products, which induce outside-in signal transduction [49]. In this regard, cell surface-bound plasmin in this study may also cleave non-ANXA2 proteins. Through the use of surface biotinylation and protease inhibitors, we found specific and reproducible changes in cleavage patterns of membrane proteins (Sato et al, unpublished results). Research on differential processing at the cell surface for the control of cellular activities may be warranted.

EPA and DHA reciprocally modulated the expression of ANXA2 on the surface of viable cells, but did not substantially affect the release of this protein bound to free membranes (Fig. 4). EPA and DHA are often classified into the same category, i.e., long-chain omega-3 PUFAs. Their effects on the responses to inflammatory cytokines agree with this conventional view [23], [24]. In the present study, however, the effect of EPA on the cell-surface bound ANXA2 may be beneficial for antagonizing the generation of plasmin on the cell surface, which may retard localized fibrinolysis *in vivo*, while an inverse outcome is anticipated for DHA. Notably, these reciprocal effects have been implicated in recent *in vivo* studies [50]–[56]. DHA levels are associated with a lower risk of arterial fibrillation [52], [53]. Administration of pure EPA is associated with a reduced risk of nonfatal coronary syndromes [54]–[56]. Since independent modulation of ANXA2 by plasmin is functioning as discussed above, attenuation of acute changes by EPA and facilitation of recovery phase of fibrinolysis by DHA may provide complimentary benefits to the vascular system.

Based on our analyses for identifying a reciprocally affected cellular mechanism, we suggest Ser-phosphorylation of ANXA2 as a target for these PUFAs (Fig. 4). Ser-phosphorylation has been shown to be mediated by PKCα, which negatively regulate the cell surface expression of ANXA2 [8]. In our study, an inhibitor of PKCα, Gö6796, blocked upregulated phosphorylation of ANXA2 by EPA (Fig. 4). PKCα binds to diacylglycerol (DAG), a lipid released by phospholipase C (PLC). In receptor signaling, two types of PLC, PLCγ1 (activated by EGFR) and PLCβ3 (activated by a Gα protein-dependent mechanism), often yield DAG through hydrolysis of PIP_2_. PIP_2_ is a preferred host phospholipid of ANXA2 *in vitro* [57]. In viable non-ECs, these molecules are distributed on the cytoplasmic face of endocytic vesicles [58] and at the interface between the plasma membrane and actin cytoskeleton [59]. We previously found that phosphorylation of Akt, which is dependent on PIP_3_ was inhibited by DHA in a breast cancer cell line [60]. Although further work is necessary, we hypothesize that DHA and EPA might reciprocally modulate the distribution of PIP_2_ and related lipids and/or its interaction with host enzyme(s). We also studied the effect of these PUFAs on Tyr-phosphorylation. With our current method, it was not clear whether EPA and DHA reciprocally induced/inhibited Tyr-phosphorylation of ANXA2. This might be because Tyr-phosphorylation is usually short-lived compared to Ser-phosphorylation even in constitutively activated cancer cells [61]. Because cell-surface bound amount of ANXA2 was small, further systematic approach for analysis of a limited fraction of proteins is necessary.

In immunofluorescence experiments, we found that ANXA2-bound caveolae relocated from cellular edges and near above the nucleus by EPA and DHA in partly different manners (Fig. 6). These fractions of ANXA2 might contain intracellular reservoir of the cell surface-bound one. In caveolae, cavolin-1 dynamically interacts with a large variety of membrane proteins, e.g., GPCRs, Gα subunit, RTKs, and cytoplasmic tyrosine kinases [62]. Entry of Ca^2+^, which is regulated by GPCRs and RTKs in endothelial cells, is robustly linked to caveolae. Loss of cavolin-1 impairs entry of Ca^2+^, and reconstitution of caveolin-1 expression rescues this defect [63], [64]. While caveolae can generally distribute over the entire cell surface, only a small and specific population responds to mechanical stimuli such as area-expanding stress [65], [66]. The distribution of EPA and DHA could change the line tension of the phospholipid bilayer. In our study, these PUFAs may have also affected the local signaling events only. This likely explains the minimal differences seen in the cell fractionation analysis of caveolae/rafts (Fig. 5).

In conclusion, we suggest that EPA and DHA may differently contribute to avoiding acute disorders of the plasminogen/plasmin system. The concentration of these PUFAs was less than 1/10th of the normal concentration of free fatty acids in the serum of healthy humans. We also suggest that proteolytic cleavage of ANXA2 is a part of the feedback regulation of this system. Further study of these mechanisms may contribute to the existing data on the nutritional support of these molecules to cardiovascular health.

## Materials and Methods

### Cells

HUV-EC-C (IFO50271, ATCC number CRL-1730) was obtained at passage of 25 from Human Science Research Resources Bank (HSRRB, Sen-nan, Osaka, Japan). The cells were cultivated at initial density of 4×10^4^/cm^2^ in medium 200 (Life Technologies, ThermoFisher, Waltham, MA) supplemented with Low Serum Growth Supplement (LSGS, consisting of 2% serum, hydrocortisone, human EGF, basic FGF and heparin, Life Technologies), 8% heat-inactivated fetal bovine serum (Hyclone, ThermoFisher) and penicillin/streptomycin (Wako Pure Chemicals, Osaka, Japan). The culture was propagated by using 0.25% trypsin/1 mM EDTA solution (Wako). They were used before passage of 33. The cells were subcultured for at least 72 h before experiments. Cell viability was assessed by measuring lactate dehydrogenase activity using a CytoTox-ONE homogeneous membrane integrity assay kit (Promega, Madison, WI).

### Antibodies and reagents

Following antibodies were obtained from indicated distributors: anti-annexin A2 (rabbit monoclonal, (CST D11G2, Danvers, MA), anti-annexin A2 (mouse monoclonal 610068, BD biosciences), anti-caveolin-1 (rabbit polyclonal Sc-894, Santa Cruz), anti-cavin-1 (anti-PTRF, rabbit polyclonal AP7421a, Abgent, San Diego, CA), anti-S100A10 (mouse monoclonal 4E7E10, CST), anti-phosphotyrosine (mouse monoclonal 4G10, Merck Millipore, Darmstadt, Germany), anti-eNOS (mouse monoclonal 610296, BD Biosciences), anti-p-Src (Y418, BS4176) and anti-p-Src (Y529, BS4729) (rabbit polyclonal, Bioworld Technology, St. Louis Park, MN), Donkey anti-rabbit IgG HRP and anti-mouse IgG HRP (Promega). OLA (Wako), EPA (Nu-Chek Prep, Elysian, MN) and DHA (Cayman Chemicals, Ann Arbor, MI) were obtained from indicate distributors. Aprotinin (Wako), α-2-antiplasmin (Haematologic Technologies Essex Junction VT), 4-(2-Aminomethyl) benzensulfonyl fluoride (AEBSF, nacalai tesque, Kyoto Japan), chymostatin (Sigma-Aldrich, St. Louis, MO) and Gö6976 (LC Laboratories, Woburn, MA) were obtained from indicated distributors. Other reagents not specified were obtained from Wako.

### Treatment of cells with fatty acids

HUV-EC-C cells were subcultured at 1×10^5^/cm^2^. After 48 h, medium was replaced by a new one without extra FBS. After additional 24 h, a dispersion of fatty acid was added at 10 µM. This was prepared by drying ethanol solutions of fatty acid (FA) followed by vortexing in the medium for 15 min at room temperature. In some experiments, the cells were pretreated with ice-cold Hank's-EGTA (Hank's balanced salt solution plus 10 mM EGTA, without Mg^2+^ and Ca^2+^, pH 7.2) at 4°C for 20 min before incubation with FAs. After culturing for 24 h, cells were washed with ice-cold TBS (10 mM Tris, 150 mM NaCl, pH 7.4). Cell surface-bound ANXA2 was extracted in Hank's-EGTA at 4°C for 20 min. It was centrifuged at 2,000×g for 5 min at 4°C. In some experiments this was further centrifuged at or 17,000×g for 10 min and 100,000×g for 1 h. For analysis of the whole proteins, the cells were scraped in TBS containing a protease inhibitor cocktail set 1 (Merck Millipore). They were immediately analyzed or stored in liquid nitrogen. Reagents were added to cells usually by using 1,000-fold concentrated solutions.

### Proteomic analysis

Proteomic analysis by two-dimensional electrophoresis (2-DE) was performed as previous described [32]. Briefly, the cells in TBS (ca. 3×10^6^ cells) were centrifuged and solubilized in a rehydration buffer which was designed for isoelectric focusing using (IEF) PROTEAN IEF Cell (Bio-Rad, Hercules, CA, USA). After centrifugation at 6,000×g for 10 min, the extracts were loaded onto ReadyStrip™ IPG Strips, pH between 3.0 and 10.0 (17 cm, Bio-Rad). IEF was performed as recommended by the manufacturer. *In situ* alkylation treatment of the gel strips for the second-dimensional SDS polyacrylamide gel electrophoresis (SDS-PAGE) was done by using a 12.5% gel. After fixation and staining by using SYPRO Ruby (Invitrogen, Carlsbad, CA), the gels were scanned for imaging using Typhoon 9400 (GE Healthcare, Buckinghamshire, UK). Peptide mass fingerprinting analysis was performed by using Autoflex II MALDI-TOF MS systems (Bruker Daltonics, Billerica, MA) as previously described [32]. Mass spectral data were used in a combined search against the NCBInr protein database with MASCOT (Matrix Science Ltd., London, UK), with parameter sets for trypsin digestion.

### Immunoblotting and immunoprecipitation

The cells and other sample solutions were solubilized in SDS-PAGE sampling buffer (containing 1% β-mercaptoethanol) by heating. For analysis of phosphoproteins, 1 mM NaF and 100 µM NaVO_4_ were included. The same amounts of cellular proteins (6 µg, determined by DC protein assay, Bio-Rad) or the same volume of cell extracts or culture supernatants were separated by SDS-PAGE using a pre-cast gel (SuperSep Ace 5–20%, Wako). Blots on PVDF sheets (immobilon-P, Merck Millipore) were blocked with 5% defatted milk or 3% bovine serum albumin (for phosphoprotein analysis) in TBS containing 0.1% Tween 20. After sequential reactions with primary and secondary antibodies, blots were visualized by using ECL prime (GE healthcare) in conjunction with LAS3000 or LAS1000 image analyzer (Fuji, Tokyo, Japan). Results were recorded as 8- or 16-bit grayscale images. Densitometric analysis was done by using ImageJ software. The differences between group means (usually n = 3) were analyzed by commercially available static analysis programs. For analysis of phosphorylated proteins immunoprecipitation, the cells were solubilized in a lysis buffer (150 mM NaCl, 1% NP-40, 0.5% deoxycholate, 50 mM Tris, 1 mM NaF, 50 mM β-glycerophosphate and 100 µM NaVO_4_ pH 8.0) containing protease inhibitors at 4°C. After overnight reaction with antibody-bound beads (Sigma), immunoprecipitate was separated as described above.

### Immunofluorescence

Glass coverslips were pretreated with medium 200 containing LSGS at 37°C for 24 h. The cells were seeded at 5×10^4^ cells/cm^2^, cultured for 72 h and used for experiments. They were fixed in PBS containing paraformaldehyde (3%) and sucrose (8%) for 10 min at room temperature. After quenching the residual aldehyde by using 50 mM glycine in PBS for 15 min, cells were permeabilized by using 50 µg digitonin (Wako) in PBS for 5 min. They were then incubated with antibodies appropriately diluted in 5% FBS-PBS in a humidified chamber at 4°C overnight. Cells were examined by double fluorescence using a rabbit antibody against caveolin-1 and a mouse mAb against ANXA2 or eNOS. Goat secondary anti-mouse or anti-rabbit IgG antibodies labeled with either AlexaFluor488 or TRITC (Invitrogen) were used for detection. Specimens were observed in an ECLIPSE E600 (Nikon, Japan) fluorescence microscope connected to a VC3000 V2 digital fine scope system (Omron, Kyoto, Japan).

### Statics

The differences between group means were analyzed by commercially available static analysis programs. Results were with p values less than 0.01.
